# Examining definitions and cues associated with alcohol‐free and low‐alcohol drinks

**DOI:** 10.1111/add.70408

**Published:** 2026-03-26

**Authors:** John Holmes, Matt Field, Colin Drummond

**Affiliations:** ^1^ School of Medicine and Population Health University of Sheffield Sheffield UK; ^2^ School of Psychology University of Sheffield Sheffield UK; ^3^ National Addiction Centre, Institute of Psychiatry, Psychology and Neuroscience King's College London London UK

*Distinguishing between alcohol‐free and low‐alcohol drinks is important for consumers but defining these categories has proved challenging. Separately, the potential for alcohol‐free drinks to change people's responses to alcohol cues may be hindered by people knowing those drinks contain no alcohol*.

We are grateful to the authors of the two responses to our article. Sinclair's commentary highlights the importance of distinguishing between alcohol‐free and low‐alcohol drinks in research, policy and clinical practice, noting our concern that few studies analyse differences between these two product types [1]. We agree on the importance of this distinction. Previous studies, as well as our own forthcoming research, find groups including pregnant women, people recovering from alcohol dependence and parents view the distinction between alcohol‐free and low‐alcohol drinks as important [2, 3, 4, 5]. The development of the no/lo drinks market in the United Kingdom (UK) also reflects this as producers launched a wide variety of unsuccessful products with lower than normal strengths before settling on alcohol‐free variants of their existing brands as the best fit with consumer demand [6].

It is more difficult to say what should count as an alcohol‐free or low‐alcohol drink. Guidance from the UK Government states only drinks up to 0.05% alcohol‐by‐volume (ABV) should be labelled as alcohol‐free [7], but this threshold is 0.5% ABV in many other countries [8]. The different definitions of alcohol‐free have little direct relevance for levels of intoxication or risks of chronic disease, but are important to people seeking to avoid consuming alcohol altogether. It is, therefore, noteworthy that the UK Government has consulted three times on changing its guidance on labelling no/lo drinks, including on proposals advanced by the alcohol industry to raise the ABV threshold for alcohol‐free drinks to 0.5%. The UK Government is yet to respond to submissions to the third consultation which ended in November 2023 [7].

Bowdring's commentary responds to concerns that the deliberately alcohol‐like properties of no/lo drinks may prompt cravings among people recovering from alcohol dependence [9]. Bowdring suggests that the same associative learning processes that lead to this ‘cue reactivity’ may, over time, extinguish the craving response if the individual remains abstinent. This is an intriguing possibility that warrants further study. Research might exploit daily diary or ecological momentary assessment designs [10] to explore if the craving response to no‐lo drink cues diminishes over time and if this extinction is robust to changes in the drinking context [11].

According to a learning theory analysis, alcohol‐related cues evoke an expectation of experiencing the subjective effects of alcohol, and this expectation plays a pivotal role in the development and continued expression of conditioned responses such as craving, attentional bias and alcohol‐seeking behaviour [12]. Consistent with learning theory, experimental findings indicate that expectancies underlie at least some aspects of reactivity to cues for alcohol and other drugs [13, 14]. This creates a challenge for Bowdring's argument because consumers of no/lo drinks are fully aware that no/lo drinks do not produce the same subjective effects as drinks containing alcohol. Therefore, one might expect any reactivity to no/lo cues to be underpinned by somewhat distinct mechanisms from those underlying reactivity to ‘real’ alcohol cues. Although this presents a challenge to the idea of using no/lo drinks to extinguish cravings associated with the expectation of consuming alcohol, it leaves in place the potential for no/lo drinks with alcohol‐like branding and appearance to elicit cue reactivity, cravings and alcohol‐seeking behaviours [15]. Exploring those mechanisms is an important direction for future experimental research.

## AUTHOR CONTRIBUTIONS

**John Holmes:** Conceptualization (lead); writing—original draft (equal). **Matt Field:** Conceptualization (equal); writing—original draft (equal). **Colin Drummond:** Writing—review and editing (supporting).

## DECLARATION OF INTERESTS

J.H. has received funding for ongoing, unrelated research on alcohol‐free and low‐alcohol drinks from Alcohol Change UK (ACUK), which received <0.6% of its funds in 2024 to 2025 from Lucky Saint, an organisation that produces and sells non‐alcoholic drinks, and owns a pub that sells standard alcoholic drinks. In March 2025, Lucky Saint became an associate member of The Portman Group, a United Kingdom self‐regulatory organisation that is fully funded and controlled by the alcohol industry. ACUK has a strict policy of not accepting any funds from, nor being subject to any influence whatsoever from, the alcohol industry, including through its investment portfolio. ACUK has confirmed that it is in full compliance with this policy.

## Data Availability

N/A.
